# An endometrial tissue-based predictive model for polycystic ovary syndrome constructed from immuno-metabolic dysregulation features mediated by ACO1

**DOI:** 10.1186/s13048-026-02036-7

**Published:** 2026-02-23

**Authors:** Peng Yi, Yangbin Qi, Suqing Mao, Ying Cao, Yanru Zhou, Xianghong Fu

**Affiliations:** 1 Center of Reproductive Medicine, The Quzhou Affiliated Hospital of Wenzhou Medical University, Quzhou People’s Hospital, Quzhou, Zhejiang 324000 China; 2 Graduate Joint Training Base, Zhejiang Chinese Medical University, Jinhua, Zhejiang 310053 China

**Keywords:** Polycystic ovary syndrome, Immuno-metabolic dysregulation, ACO1, Predictive model, Machine learning

## Abstract

**Objective:**

Polycystic ovary syndrome (PCOS) is a multifactorial endocrine disorder characterized by reproductive and metabolic abnormalities. This study aimed to identify key immunometabolic regulators in endometrial tissue and construct a predictive model for PCOS using machine learning approaches.

**Methods:**

Three endometrial transcriptomic datasets (GSE277906, GSE193123, GSE199225) were integrated and analyzed for differentially expressed genes (DEGs), immune cell infiltration, and metabolic pathway enrichment. Core genes were identified via protein–protein interaction networks and functional annotation. A predictive model was developed using SVM-RFE, XGBoost, and random forest algorithms and validated through qRT-PCR on granulosa cell samples.

**Results:**

Five core metabolism-related genes were identified, among which ACO1 was consistently downregulated and negatively correlated with CD8⁺ T cell infiltration. High ACO1 expression was enriched in oxidative phosphorylation and mTOR signaling, while low expression was associated with immune activation. The random forest model incorporating ACO1, CHPF, and STOML1 achieved strong predictive performance (AUC = 0.800).

**Discussion:**

ACO1 may function as an immunometabolic modulator by linking iron metabolism, oxidative stress, and T cell activity. Its downregulation may contribute to local immune suppression and endometrial dysfunction in PCOS. The tissue-level model demonstrated good diagnostic value and biological interpretability across cohorts.

**Conclusion:**

This study highlights ACO1 as a key biomarker of immunometabolic dysregulation in PCOS and presents a robust predictive model for early diagnosis. The findings offer new insights into the molecular mechanisms underlying PCOS and suggest potential targets for precision treatment.

**Supplementary Information:**

The online version contains supplementary material available at 10.1186/s13048-026-02036-7.

## Introduction

Polycystic ovary syndrome (PCOS) is a multisystem disorder primarily characterized by ovarian dysfunction, affecting over 11% of reproductive-aged women worldwide [1]. Its typical clinical manifestations include hyperandrogenism, ovulatory dysfunction, and polycystic ovarian morphology, often accompanied by metabolic abnormalities such as insulin resistance, dyslipidemia, and obesity [2]. These factors significantly increase the risk of developing type 2 diabetes, cardiovascular disease, and endometrial cancer [3]. In recent years, PCOS has been increasingly recognized as a complex systemic condition driven by dysregulated immunometabolic interactions, extending beyond the traditional paradigm of endocrine dysfunction [4].

Accumulating evidence has revealed profound remodeling of the immune microenvironment in PCOS, particularly involving imbalances in T cell subsets, macrophage polarization anomalies, and increased activation of natural killer (NK) cells ([5, 6]. These immune alterations not only manifest peripherally but also directly impact the functional integrity of the endometrium, contributing to disrupted implantation windows and impaired follicular development [7]. Notably, metabolic pathways and immune cell functions are tightly intertwined; aberrations in processes such as ferroptosis, mTOR signaling, and lipid metabolism have been shown to influence T cell exhaustion, antigen responsiveness, and memory formation [8, 9]. However, integrated studies combining immune cell infiltration profiling, metabolism-related gene expression, and tissue-level transcriptomic data in PCOS are still lacking. Such analyses are essential to identify core molecular regulators that underpin systemic immunometabolic dysfunction in this disorder. In recent years, integrative multi-omics studies combining transcriptomic data with immune and metabolic features have begun to characterize the systemic immunometabolic landscape of PCOS, identifying key inflammatory and metabolic regulators at the tissue and cellular levels [10]. In parallel, emerging single-cell transcriptomic analyses have provided higher-resolution views of ovarian cell-type heterogeneity and developmental trajectories associated with PCOS [11].

In parallel, machine learning algorithms based on high-throughput omics data have shown promise in feature selection and risk prediction across complex diseases, offering strong interpretability and predictive accuracy [12]. While some models have been developed in PCOS using serum hormones, metabolic indices, or circulating RNAs [13], these approaches predominantly focus on systemic peripheral markers and have yet to fully explore the biological information embedded in the local immunometabolic landscape of the endometrium. Furthermore, existing models often exhibit limited robustness and biological interpretability across cohorts, restricting their applicability in precision medicine. 

To address these gaps, we systematically integrated multiple endometrial transcriptomic datasets related to PCOS from the GEO database. After batch correction and normalization, we performed differential expression analysis and immune infiltration profiling, coupled with metabolic pathway enrichment and protein–protein interaction network analysis, to identify key candidate genes. Based on these genes, we constructed multigene prediction models using support vector machine recursive feature elimination (SVM-RFE), XGBoost, and random forest algorithms. We further validated their expression stability and predictive value using qRT-PCR experiments. The aims of this study were to: (1) identify core regulators associated with immunometabolic dysregulation, particularly those involved in T cell–related pathways; (2) develop a tissue-level predictive model with cross-cohort robustness; and (3) provide theoretical insights and potential biomarkers for molecular subtyping and targeted intervention in PCOS.

## Materials and methods

### Data collection and preprocessing

Three gene expression datasets related to polycystic ovary syndrome (PCOS) in human endometrial tissues—GSE277906 (RNA-sequencing, GPL24676), GSE193123 (microarray, GPL570), and GSE199225 (microarray, GPL17077)—were retrieved from the Gene Expression Omnibus (GEO) database. Raw expression data were preprocessed according to the corresponding platform annotation files. For microarray datasets, background correction and quantile normalization were performed, followed by log₂ transformation. For RNA-sequencing data, normalized expression matrices provided by the GEO database were directly used for downstream analysis. GSE277906 and GSE193123 were merged to form the training cohort, and batch effect correction was performed using the ComBat function implemented in the sva R package (version 3.46.0). After correction, principal component analysis (PCA) was conducted to evaluate sample distribution and confirm distinguishable expression profiles between groups.

### Differential expression analysis and functional enrichment

Differential expression analysis between PCOS and control samples in the training set was performed using the limma R package (version 3.58.1), with significance thresholds set at |log₂ fold change| > 1 and *p* < 0.05. The resulting differentially expressed genes (DEGs) were subjected to Gene Ontology (GO) and Kyoto Encyclopedia of Genes and Genomes (KEGG) enrichment analysis using the ClusterProfiler package (version 4.10.0). GO terms were categorized into biological process (BP), molecular function (MF), and cellular component (CC) levels.

### Protein–protein interaction (PPI) network and hub gene identification

All identified DEGs were submitted to the STRING database (v11.5) to construct a protein–protein interaction (PPI) network. The resulting network was visualized using Cytoscape (v3.9.1). The cytoHubba plugin was used to calculate node centrality based on degree and MCC (Maximal Clique Centrality) algorithms, in order to identify potential hub genes.

### Immune cell infiltration analysis and metabolic pathway profiling

Immune cell infiltration in the GSE199225 validation dataset was estimated using the CIBERSORT algorithm with the LM22 signature matrix, and only samples with CIBERSORT output p values < 0.05 were retained for subsequent analysis, covering 22 immune cell subtypes. Differences in immune composition between PCOS and control samples were compared, and Spearman correlation analysis was performed to examine associations between candidate hub gene expression and the abundance of key immune cells, such as CD8⁺ T cells.

In addition, based on the expression levels of selected core genes, samples were divided into high- and low-expression groups. Single-sample gene set enrichment analysis (ssGSEA) was conducted using the GSVA R package (version 1.50.0) to evaluate the enrichment of relevant immune and metabolic pathways in each group.

### Core gene selection and machine learning model construction

After intersecting DEGs from the training set with a predefined list of metabolism-related genes, five core candidate genes—including ACO1, CHPF, and STOML1—were identified. Three supervised machine learning classification models were developed based on the selected genes: (1) Support Vector Machine–Recursive Feature Elimination (SVM-RFE); (2) Extreme Gradient Boosting (XGBoost); (3) Random Forest (RF).

Model training and parameter optimization were conducted using the caret R package (version 6.0–94). The merged datasets (GSE277906 and GSE193123) were used as the training cohort, while GSE199225 served as an independent validation cohort. Model training and hyperparameter tuning were performed using k-fold cross-validation. To ensure reproducibility, a fixed random seed was applied throughout the model training and evaluation process. The classification performance of each model was assessed using receiver operating characteristic (ROC) curves and area under the curve (AUC) values in both training and independent validation datasets.

### Clinical sample collection and granulosa cell isolation

Granulosa cell samples were obtained from women with PCOS (*n* = 3) and age-matched controls without PCOS (*n* = 3) undergoing in vitro fertilization (IVF) at the Center of Reproductive Medicine, The Quzhou Affiliated Hospital of Wenzhou Medical University. PCOS was diagnosed according to the Rotterdam 2003 criteria, defined by the presence of at least two of the following features: clinical and/or biochemical hyperandrogenism, ovulatory dysfunction, and polycystic ovarian morphology, after exclusion of other endocrine disorders. Women without PCOS and with a comparable age distribution to the PCOS group were enrolled as controls. To minimize potential confounding effects, participants with body mass index (BMI) > 28 kg/m², basal follicle-stimulating hormone (FSH) level > 10 mIU/mL, endometriosis, diminished ovarian reserve, chromosomal abnormalities, hydrosalpinx, systemic illness, or endocrine disorders were excluded. All participants provided written informed consent. The study protocol was approved by the Ethics Committee of The Quzhou Affiliated Hospital of Wenzhou Medical University (Quzhou People’s Hospital) (approval number: 2024-061), and all procedures involving human participants were conducted in accordance with the Declaration of Helsinki.

On the day of oocyte retrieval, clear follicular fluid containing granulosa cells was collected during transvaginal ultrasound-guided follicular aspiration. Granulosa cells were isolated from follicular fluid by centrifugation and enzymatic dispersion. The isolated granulosa cells were washed with phosphate-buffered saline (PBS) and stored at − 80 °C for subsequent RNA extraction.

### Experimental validation via quantitative real-time PCR (qRT-PCR)

**Fig. 1:**
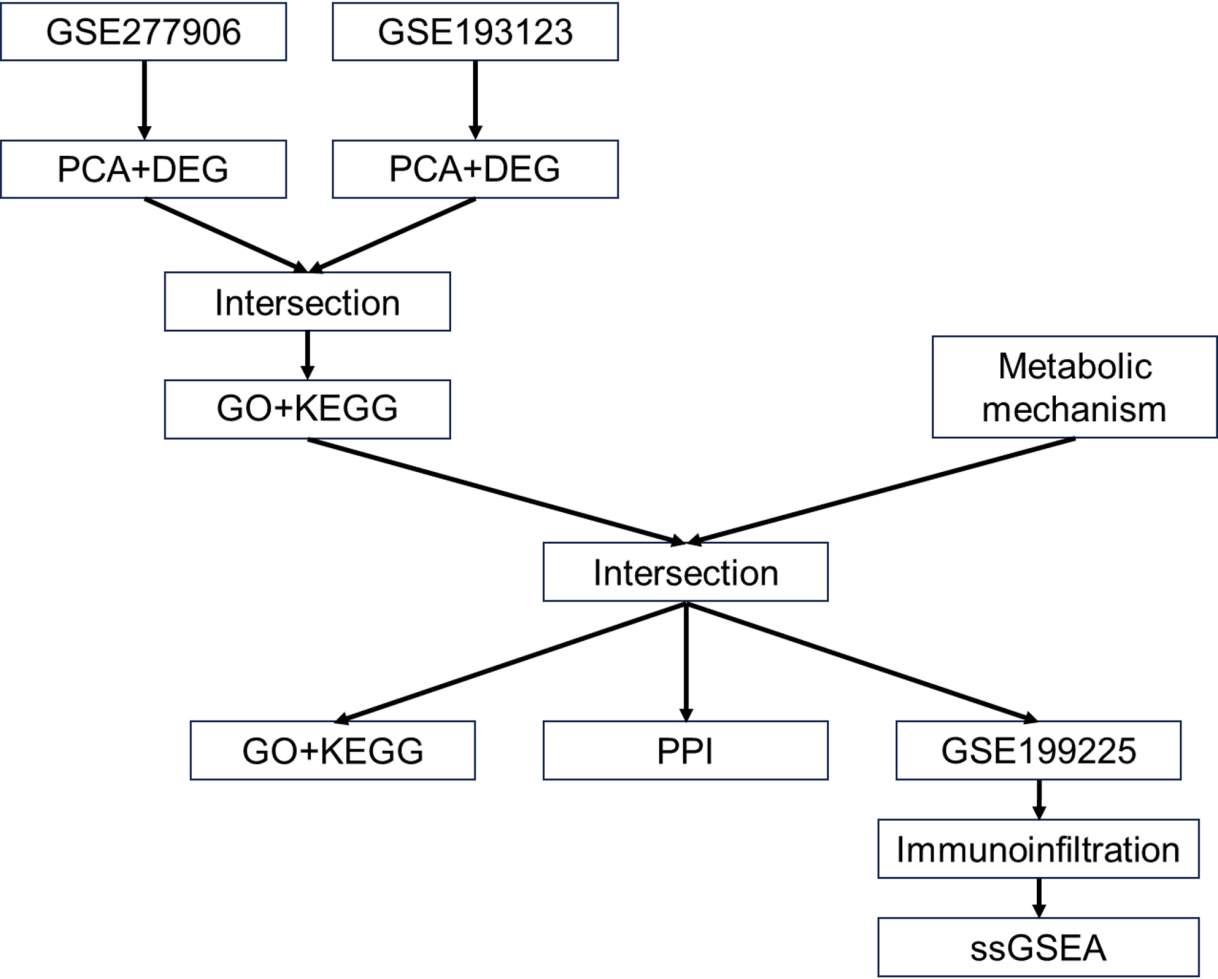
Study design and analysis workflow. Differentially expressed genes (DEGs) were identified from GSE277906 and GSE193123, followed by intersection and GO/KEGG enrichment. Core metabolic genes were screened through pathway analysis and PPI network construction. GSE199225 was used for immune infiltration analysis and ssGSEA. Key immune–metabolic genes were integrated for model construction

Total RNA was extracted using TriPure reagent and quantified using a NanoDrop™ One spectrophotometer. cDNA synthesis was performed with the High-Capacity RNA-to-cDNA™ Kit, and qRT-PCR was carried out using Fast SYBR™ Green Master Mix on an Applied Biosystems 7300 Real-Time PCR System. Each sample was analyzed in triplicate. B2M and ACTB were used as internal controls, and relative gene expression levels were calculated using the 2⁻ΔΔCT method. The primer sequences are listed in Supplementary Table S1. The workflow of this study is illustrated in Fig. 1.

## Results

### Sample integration, differential expression analysis, and metabolic pathway enrichment

**Fig. 2:**
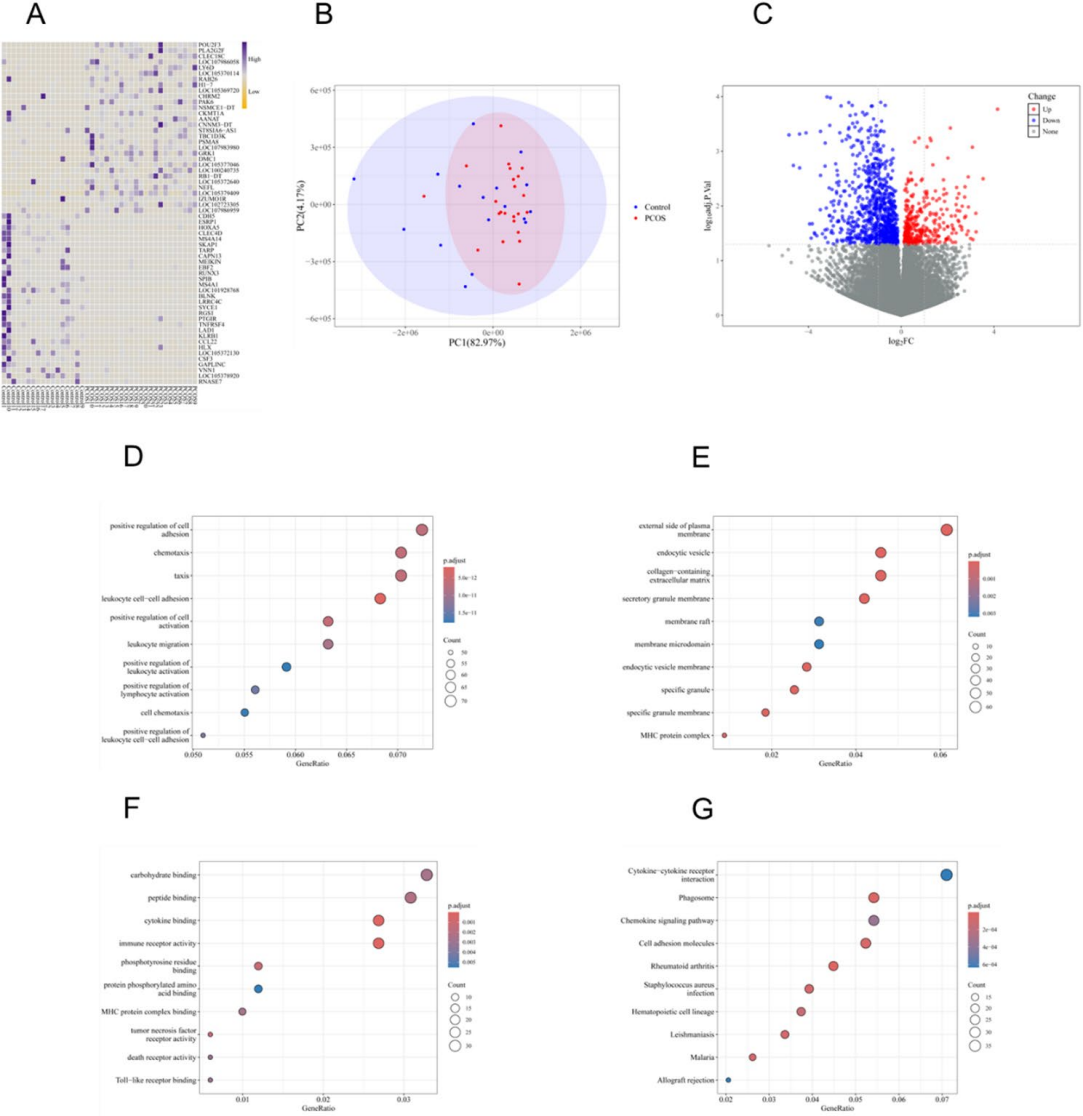
Transcriptomic analysis and pathway enrichment of PCOS and control endometrial samples. **A** Heatmap of differentially expressed genes (DEGs) in the training dataset. **B** Principal component analysis (PCA) plot of PCOS and control samples after batch effect correction. **C** Volcano plot of DEGs identified by limma analysis (|log₂FC| > 1, p < 0.05). **D**–**F** Gene Ontology (GO) enrichment analysis of DEGs, including biological process (BP), cellular component (CC), and molecular function (MF). **G** Kyoto Encyclopedia of Genes and Genomes (KEGG) pathway enrichment analysis of DEGs

In this study, two training datasets—GSE277906 (23 PCOS samples, 17 controls) and GSE193123 (3 PCOS samples, 3 controls)—were integrated. Batch effects were corrected using the ComBat method, and principal component analysis (PCA) confirmed good alignment between datasets, with clear group-level clustering (Fig. 2B). Differential expression analysis was conducted using the limma package, with thresholds set at |log₂FC| > 1 and *p* < 0.05, identifying multiple genes significantly differentially expressed between PCOS and control groups (Fig. 2C). A heatmap further illustrated the distinct expression patterns of these differentially expressed genes (DEGs) across samples (Fig. 2A).

**Fig. 3:**
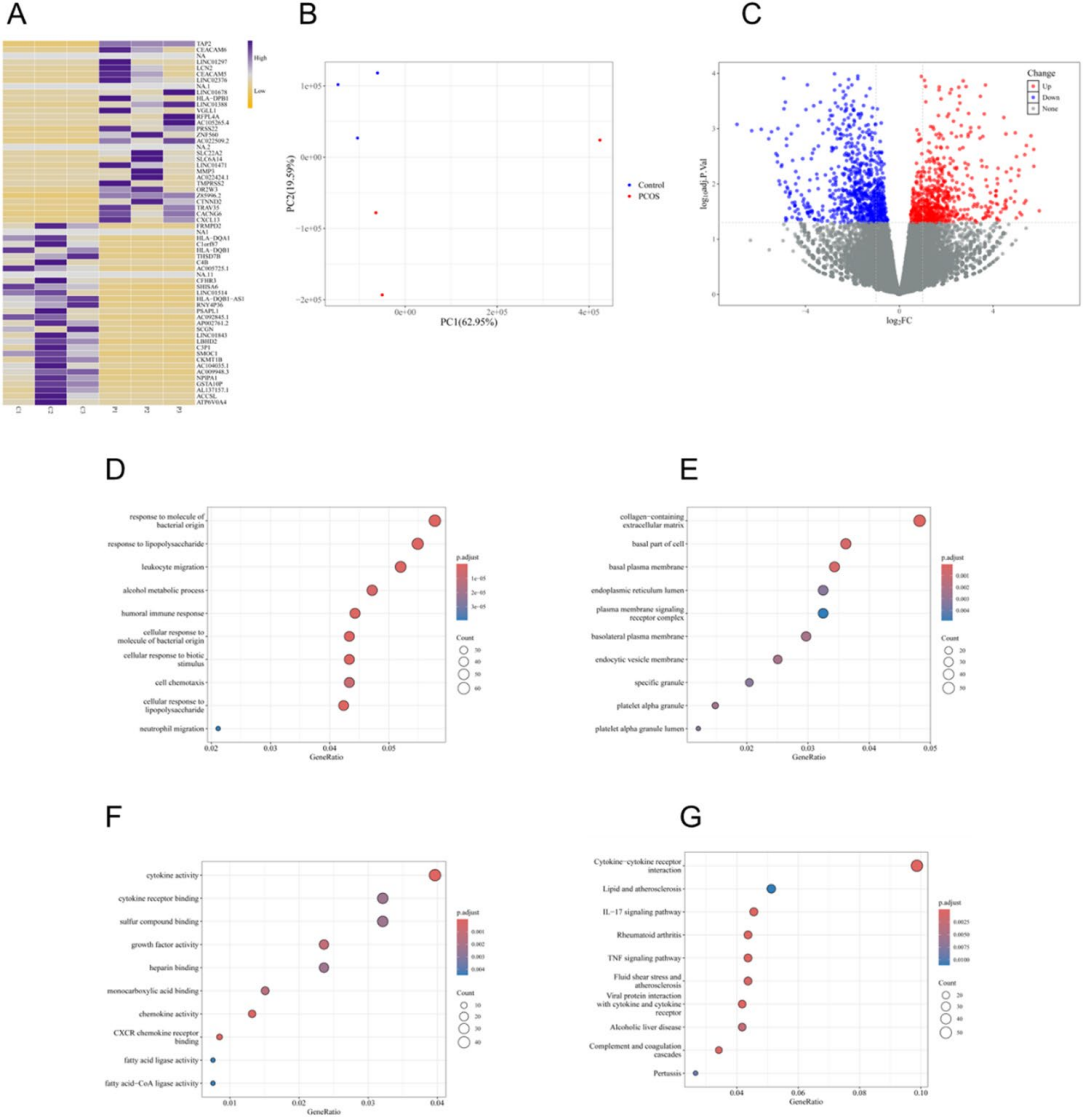
Differential expression and functional enrichment analysis of endometrial samples from GSE193123. **A** Heatmap of differentially expressed genes (DEGs) between PCOS and control samples. **B** Principal component analysis (PCA) plot of PCOS and control samples. **C** Volcano plot of DEGs identified by limma analysis (|log₂FC| > 1, p < 0.05). **D**–**F** Gene Ontology (GO) enrichment analysis of DEGs, including biological process (BP), cellular component (CC), and molecular function (MF). **G** Kyoto Encyclopedia of Genes and Genomes (KEGG) pathway enrichment analysis of DEGs

Functional annotation and pathway enrichment of these DEGs revealed significant enrichment in key metabolic biological processes, including lipid metabolism, iron ion homeostasis, and redox regulation, as identified by Gene Ontology (GO) analysis (Fig. 2D–F). Kyoto Encyclopedia of Genes and Genomes (KEGG) pathway analysis further indicated that these genes are involved in critical metabolic networks such as the tricarboxylic acid (TCA) cycle, fatty acid degradation, oxidative phosphorylation, and the mTOR signaling pathway (Fig. 2G). Similar expression patterns and pathway enrichment trends were observed in the GSE193123 dataset (Fig. 3A–G), confirming the consistency of these findings across cohorts and indicating the presence of systemic metabolic dysregulation in the endometrial tissue of PCOS patients.

### Core metabolic gene selection and immune infiltration characterization

**Fig. 4:**
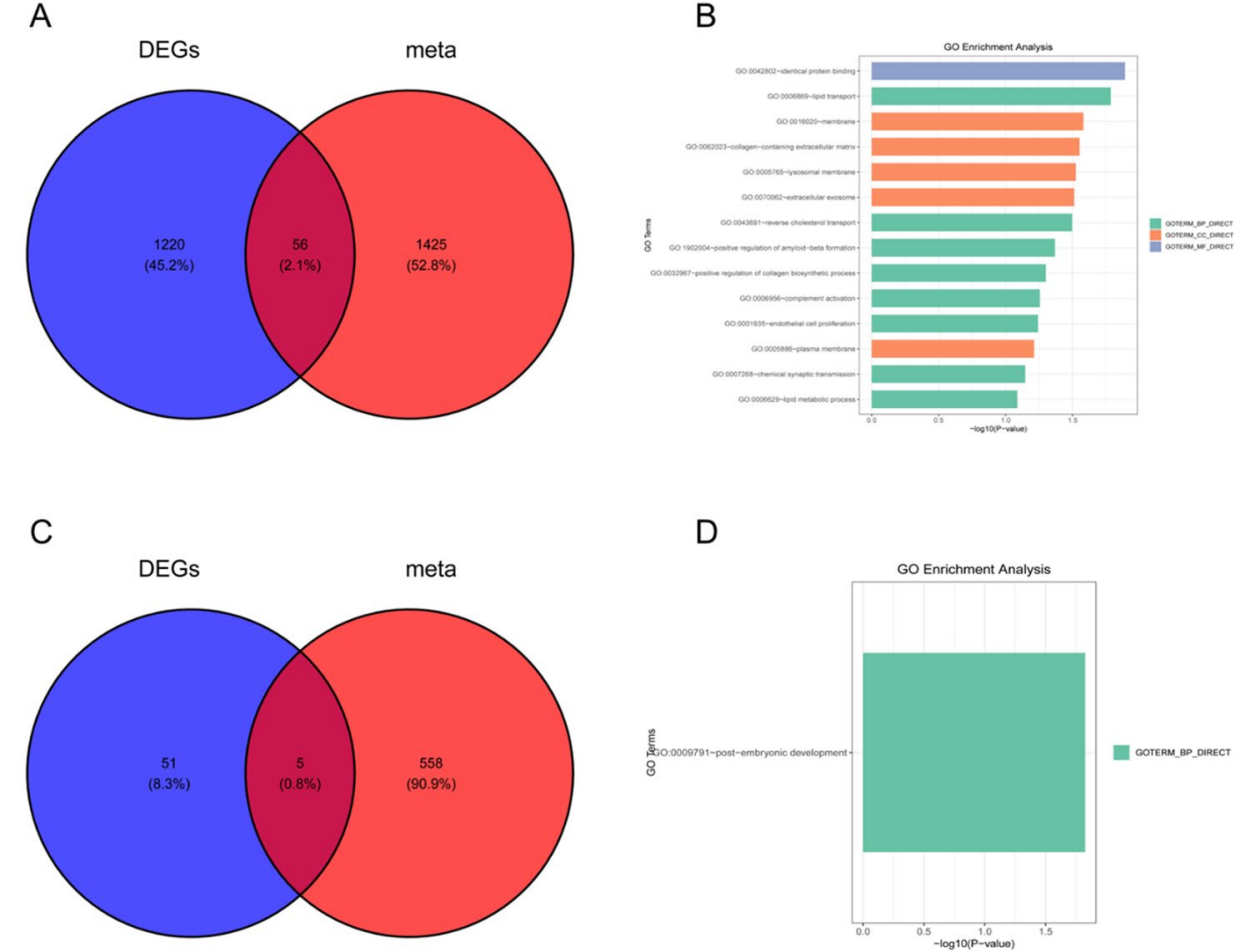
Identification and functional annotation of metabolism-related core genes in PCOS. **A **Venn diagram showing the intersection between differentially expressed genes (DEGs) and metabolism-related genes, resulting in 56 overlapping candidates.;(**B**) GO enrichment analysis of the 56 overlapping genes. The genes were significantly enriched in biological processes including metal ion binding, extracellular matrix organization, and transmembrane transporter activity.;(**C**) Further filtering of the 56 genes by KEGG pathway association and metabolic function yielded 5 core metabolism-related genes (including ACO1, CHPF, and STOML1), as shown in the second Venn diagram.;(**D**) GO enrichment analysis of the final 5 core genes, highlighting their involvement in post-embryonic development and extracellular structural organization

After integrating the DEGs from the two training datasets, we intersected them with a curated metabolic gene set and identified five core candidate genes, including ACO1, CHPF, and STOML1 (Fig. 4A–C). GO enrichment analysis revealed that these genes are predominantly involved in metal ion binding, transmembrane transport, and extracellular matrix organization, suggesting their potential roles in maintaining cellular homeostasis and tissue structural integrity (Fig. 4D).

**Fig. 5:**
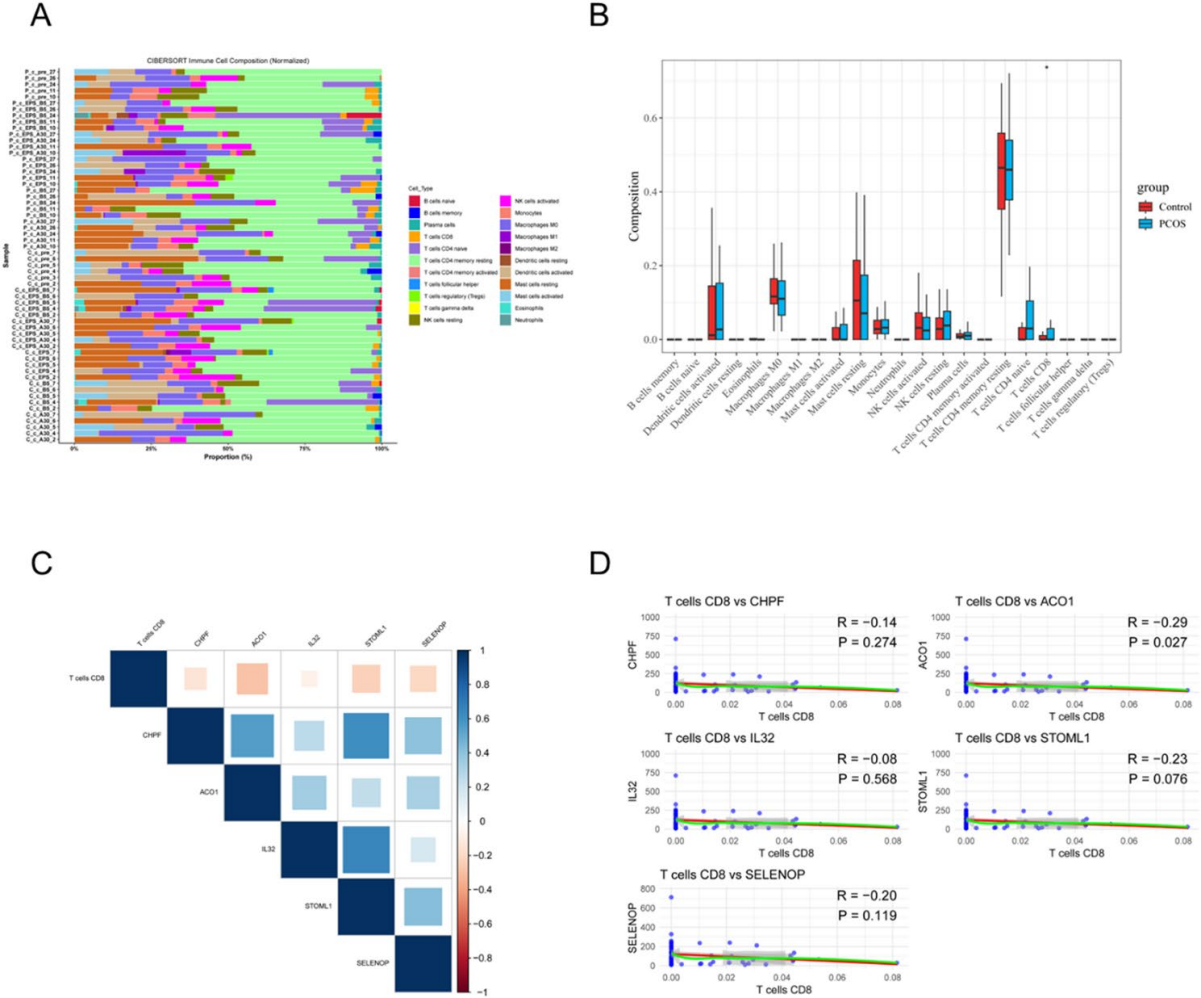
Immune cell infiltration landscape and correlation with core metabolic genes in PCOS. **A** Bar plot showing the relative proportions of 22 immune cell subtypes in each sample of the GSE199225 dataset, as estimated by the CIBERSORT algorithm. Differences in immune cell composition between PCOS and control groups are visualized.;(**B**) Boxplot comparing the relative abundance of selected immune cell types between PCOS and control groups. CD8⁺ T cells were significantly reduced in PCOS samples, while M1 macrophages and activated NK cells showed a mild increase.;(**C**) Correlation heatmap depicting the Spearman correlation coefficients between CD8⁺ T cells and the five core metabolic genes (ACO1, CHPF, STOML1, IL32, and SELENOP). Stronger negative associations are shown in darker shades of blue.;(**D**) Scatter plots showing individual correlations between CD8⁺ T cell infiltration levels and the expression of each core gene. Notably, ACO1 expression was negatively correlated with CD8⁺ T cells (R = –0.47, p = 0.027), suggesting its potential role in immune regulation

Immune infiltration analysis was performed using the CIBERSORT algorithm on the independent validation dataset GSE199225. The results showed that CD8⁺ T cell proportions were significantly reduced in PCOS samples, while M1 macrophages and activated NK cells exhibited slight increases (Fig. 5A–B). Further Spearman correlation analysis revealed a significant negative correlation between ACO1 expression and CD8⁺ T cell infiltration (*r* = − 0.47, *p* < 0.05) (Fig. 5C–D), suggesting that ACO1 may play a role in modulating T cell recruitment or function, thereby contributing to local immune microenvironment remodeling.

To assess the centrality of these core genes within protein interaction networks, we constructed a protein–protein interaction (PPI) network using the STRING database, and key hub genes were identified with the cytoHubba plugin in Cytoscape. ACO1, CHPF, and STOML1 were located in the highly interconnected central modules of the network, forming a potential immunometabolic regulatory hub (Figure S1).

### ACO1 expression-associated immunometabolic pathway characteristics

Based on ACO1 expression levels, samples were stratified into high-expression and low-expression groups, followed by single-sample gene set enrichment analysis (ssGSEA) (Figure S2). The high ACO1 expression group was significantly enriched in oxidative phosphorylation, lipid biosynthesis, mTOR signaling, and ferroptosis-related metabolic pathways, whereas the low expression group showed enrichment in immune activation, antigen processing, and chemokine signaling pathways. This expression-function dichotomy suggests that ACO1 may act as an immunometabolic coupling factor, potentially modulating the inflammatory and metabolic state of PCOS endometrial tissue.

### Machine learning model construction and predictive performance evaluation

Using the identified core genes, we constructed three classification models: support vector machine with recursive feature elimination (SVM-RFE), XGBoost, and random forest. Among them, the random forest model achieved the best performance in the training set (AUC = 0.875) and retained strong discriminative ability in the independent validation cohort (AUC = 0.800), demonstrating good generalizability (Figure S3A). Additional performance metrics, including precision, recall, and F1-score, are summarized in Supplementary Table S2. Notably, ACO1 consistently ranked as one of the most important features across all models, supporting its role as a key explanatory and predictive biomarker. The feature importance ranking derived from the random forest model is provided in Supplementary Table S3. In the GSE199225 validation dataset, ACO1 expression was significantly lower in the PCOS group compared to controls (*p* = 0.044). Although CHPF and STOML1 did not reach statistical significance, their expression trends were consistent with those observed in the training set (Figure S3B), further validating ACO1 as a stable diagnostic marker across cohorts.

### qRT-PCR validation of candidate gene expression

To experimentally validate the core candidate genes, qRT-PCR was performed on ovarian granulosa cell samples obtained from clinical PCOS and control patients. The results showed that ACO1, CHPF, STOML1, and SELENOP were significantly downregulated, while IL32 was significantly upregulated in the PCOS group (Figure S4). The expression trend of ACO1 was consistent across the transcriptomic datasets and the qRT-PCR experiments, further confirming its robust regulatory role in PCOS.

## Discussion

In this study, we integrated multiple transcriptomic datasets of endometrial tissue from patients with polycystic ovary syndrome (PCOS), combined immune infiltration analysis, and applied machine learning algorithms to identify core candidate genes closely related to metabolic dysregulation. We further constructed a multi-gene prediction model that demonstrated favorable diagnostic performance. Among the identified genes, ACO1 consistently exhibited downregulated expression across datasets and showed a significant negative correlation with CD8⁺ T cell infiltration, suggesting its potential central role in immunometabolic regulation in PCOS.

Our ssGSEA results revealed that samples with high ACO1 expression were significantly enriched in mTOR signaling, oxidative phosphorylation, lipid biosynthesis, and ferroptosis-related pathways, whereas low ACO1 expression samples were enriched in immune activation, antigen processing, and chemokine signaling pathways. Previous studies have demonstrated that ACO1 not only functions in the tricarboxylic acid (TCA) cycle but also converts to iron regulatory protein 1 (IRP1) under low intracellular iron conditions, modulating iron homeostasis genes and influencing oxidative stress and mitochondrial function [14]. Based on our transcriptomic associations and immune infiltration analyses, we hypothesize that ACO1 downregulation may be involved in perturbing an iron homeostasis–mTOR–T cell metabolic axis; however, this proposed regulatory link remains speculative in the absence of direct functional validation. This aligns with recent reports highlighting the role of ferroptosis in granulosa cell dysfunction and PCOS pathogenesis [8]. ACO1 may thus serve as a molecular bridge between metabolic and immune dysregulation, representing a hallmark feature of metabolically driven PCOS.

Consistent with previous single-cell transcriptomic studies by Eriksson et al. [4], we observed a significant reduction in CD8⁺ T cells in the endometrial tissue of PCOS patients, indicating T cell hypoactivation and local immune imbalance as contributors to endometrial dysfunction. Beyond CD8⁺ T cell depletion, our immune infiltration analysis also revealed modest increases in activated natural killer (NK) cells and M1 macrophages, suggesting a broader reshaping of the local immune microenvironment rather than uniform immune suppression. These immune alterations are closely linked to metabolic cues, including lipid metabolism, oxidative stress, and iron homeostasis, which are known to be disrupted in PCOS and may collectively contribute to a low-grade inflammatory milieu accompanied by impaired T cell responsiveness. Importantly, local tissue-level immune alterations may not fully mirror systemic inflammatory changes observed in PCOS. For instance, Zorlu et al. reported that peripheral inflammatory indices, including the delta neutrophil index, were associated with clinical and sonographic features of PCOS, highlighting the contribution of systemic immune activation [15]. Together, these findings suggest that PCOS is characterized by compartment-specific immune dysregulation, in which endometrial immune remodeling differs from peripheral inflammatory signatures, underscoring the value of tissue-based immunometabolic analyses for understanding PCOS pathophysiology.

The observed inverse association between ACO1 expression and CD8⁺ T cell infiltration indicates a close relationship between ACO1-related metabolic states and local T cell alterations in the endometrium, raising the possibility of an immunometabolic link without implying direct functional causality. Such immunometabolic dysregulation within the endometrium may have clinically relevant consequences, as coordinated immune and metabolic homeostasis is essential for maintaining endometrial receptivity and a functional implantation window in women with PCOS. Although reproductive outcomes were not directly assessed in this study, our findings suggest that ACO1-associated immune and metabolic imbalance may represent a potential mechanistic link between PCOS-related endometrial dysfunction and infertility risk. As T cells play an essential role in the implantation window and endometrial receptivity, their depletion in PCOS may not only compromise immune homeostasis but also contribute to infertility risk [7].

We developed classification models based on SVM-RFE, XGBoost, and random forest algorithms, using ACO1, CHPF, and STOML1 as core features. Among them, the random forest model achieved the highest performance in the training set and maintained strong generalizability in the independent validation cohort (AUC = 0.800). Unlike traditional diagnostic approaches based on serum hormone levels or peripheral markers, our model integrates tissue-specific transcriptomic features and immunometabolic signatures, offering enhanced biological interpretability and mechanistic relevance. From a clinical perspective, this tissue-based model may be most applicable to reproductive-aged women with suspected or infertility-associated PCOS, particularly those undergoing infertility evaluation or assisted reproductive procedures. Endometrial tissue obtained during routine diagnostic biopsy or fertility-related clinical interventions could serve as a feasible sampling source, supporting the model’s use as an adjunctive tool. Previous transcriptome-based machine learning studies in PCOS have mainly focused on diagnostic biomarker screening using non-endometrial transcriptomic data. For example, Chen et al. constructed machine learning models based on granulosa cell RNA-seq data by integrating LASSO and SVM-RFE algorithms, and further explored immune cell infiltration patterns using CIBERSORT to identify potential diagnostic hub genes [16]. In this context, He et al. employed integrated transcriptomics and machine learning to identify shared diagnostic genes and potential mechanisms between PCOS and recurrent miscarriage, highlighting the broader applicability of transcriptome-driven modeling in uncovering disease-associated molecular networks [17]. However, these studies primarily emphasized cross-condition gene prioritization and pathway-level interpretation, rather than tissue-specific immune–metabolic integration within a defined diagnostic framework. In contrast, the present study specifically focuses on endometrial tissue and integrates immune infiltration profiles, metabolism-related gene signatures, and tissue-level transcriptomic data into a unified modeling strategy, thereby enhancing biological interpretability and relevance to local endometrial dysfunction in PCOS.

While several existing PCOS biomarker models focus on circulating RNA or miRNA signatures [13], this study, to our knowledge, is the first to propose a local tissue-based predictive model from an immunometabolic perspective, offering new avenues for molecular subtyping and targeted therapeutic interventions in PCOS.

Despite the multi-dimensional strengths of our analytical framework, this study has several limitations. First, some of the public datasets included relatively small sample sizes, which may introduce inter-cohort heterogeneity and limit statistical power. Second, immune cell infiltration was inferred using the CIBERSORT algorithm with the LM22 signature, which is derived from peripheral blood mononuclear cells and therefore provides only an approximate estimation of immune cell composition in endometrial tissue, rendering the observed immune alterations correlative and requiring orthogonal validation in future studies. Third, although qRT-PCR validated the differential expression of ACO1, CHPF, and STOML1 at the transcript level, confirmation at the protein level and functional validation will be necessary to establish a causal relationship. Fourth, granulosa cells were used for qRT-PCR validation mainly because of their clinical accessibility and their close involvement in PCOS-related metabolic and inflammatory dysregulation; however, since transcriptomic discovery and model construction were based on endometrial tissue datasets, this difference in tissue context represents an important limitation. Consequently, the qRT-PCR findings should be interpreted as supportive cross-tissue evidence rather than direct validation at the endometrial tissue level.

Future research should focus on delineating the role of ACO1 in T cell subset regulation, oxidative stress responses, and iron metabolism signaling. Integrating single-cell and spatial transcriptomics will further elucidate ACO1’s spatial dynamics along the ovary–uterus–immune axis. Moreover, combining transcriptomic data with peripheral biomarkers, metabolic profiles, and epigenomic features may support the development of personalized diagnostic and therapeutic strategies for PCOS.

## Conclusion

By integrating multiple endometrial transcriptomic datasets related to PCOS, we identified ACO1, CHPF, and STOML1 as core metabolism-related genes and demonstrated a significant negative correlation between ACO1 expression and CD8⁺ T cell infiltration. The multi-gene machine learning model we developed achieved strong performance in an independent validation cohort (AUC = 0.800), with robust stability and generalizability. These findings uncover a critical molecular link between local immunometabolic imbalance and PCOS pathophysiology, and highlight ACO1 as a potential biomarker for early diagnosis, molecular subtyping, and targeted treatment of metabolically driven PCOS, with meaningful clinical and public health implications.

## Supplementary Information

Supplementary Material 1: Figure S1. Protein–protein interaction (PPI) network of core candidate genes. The PPI network was constructed based on the STRING database to visualize interactions among the core candidate genes and their associated proteins. Nodes represent proteins, and edges represent protein–protein interactions. Edge colors indicate types of interaction evidence, while node size and edge thickness reflect interaction strength. ACO1, CHPF, STOML1, IL32, and SELENOP were positioned at central hubs in the network, suggesting their key regulatory roles in immunometabolic modulation.

Supplementary Material 2: Figure S2. GO enrichment and ssGSEA pathway analysis associated with ACO1 expression. (A) Bar plot of GO terms enriched in the low ACO1 expression group, mainly associated with extracellular matrix organization and structural components.;(B) Bar plot of GO terms enriched in the high ACO1 expression group, related to inflammatory cytokine response, chemotaxis, and immune activation.;(C) Bubble plot of GO enrichment for the low ACO1 group, showing significant enrichment in metabolic and structural pathways.;(D) Bubble plot of GO enrichment for the high ACO1 group, highlighting immune-related processes including granulocyte migration and cytokine-mediated signaling.

Supplementary Material 3: Figure S3. Expression levels of core genes and model performance evaluation. (A) Receiver operating characteristic (ROC) curves and area under the curve (AUC) values of three machine learning models (SVM-RFE, Random Forest, XGBoost) in the validation cohort. The Random Forest model achieved the highest performance (AUC = 0.800).;(B) Box plots of log₂-transformed expression levels of ACO1, CHPF, and STOML1 in PCOS versus control samples. ACO1 showed a statistically significant difference between groups (p = 0.044).

Supplementary Material 4: Figure S4. qRT-PCR validation of core gene expression in clinical samples. Quantitative real-time PCR (qRT-PCR) was used to measure mRNA levels of the five core genes in granulosa cells from PCOS and control patients. CHPF, SELENOP, ACO1, and STOML1 were significantly downregulated in the PCOS group, while IL32 was significantly upregulated, consistent with transcriptomic findings.

Supplementary Material 5: Supplementary Tables.

## Data Availability

The data used to support the findings of this study are available from the corresponding author upon request.
