# Supplementary figures and images for: An endometrial tissue-based predictive model for polycystic ovary syndrome constructed from immuno-metabolic dysregulation features mediated by ACO1
Source: J Ovarian Res. 2026 Feb 23;19:121. doi: 10.1186/s13048-026-02036-7 (PMC13036953; doi:10.1186/s13048-026-02036-7)

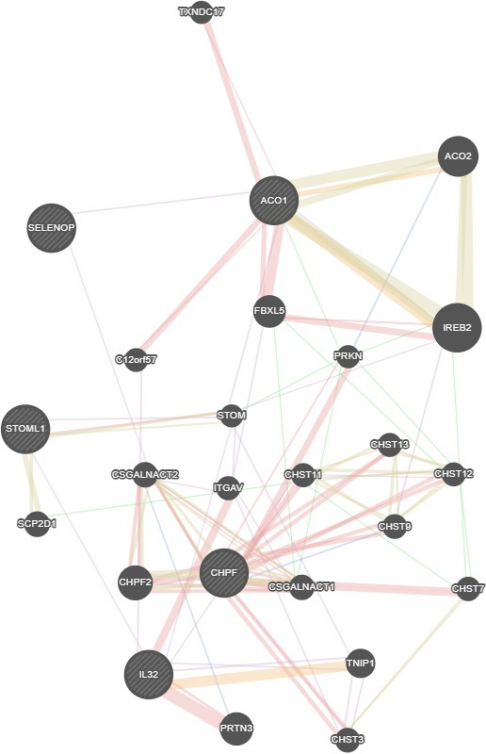

Supplement: Supplementary file 1 — Supplementary Material 1: Figure S1. Protein–protein interaction (PPI) network of core candidate genes. The PPI network was constructed based on the STRING database to visualize interactions among the core candidate genes and their associated proteins. Nodes represent proteins, and edges represent protein–protein interactions. Edge colors indicate types of interaction evidence, while node size and edge thickness reflect interaction strength. ACO1, CHPF, STOML1, IL32, and SELENOP were positioned at central hubs in the network, suggesting their key regulatory roles in immunometabolic modulation. [file 13048_2026_2036_MOESM1_ESM.png]

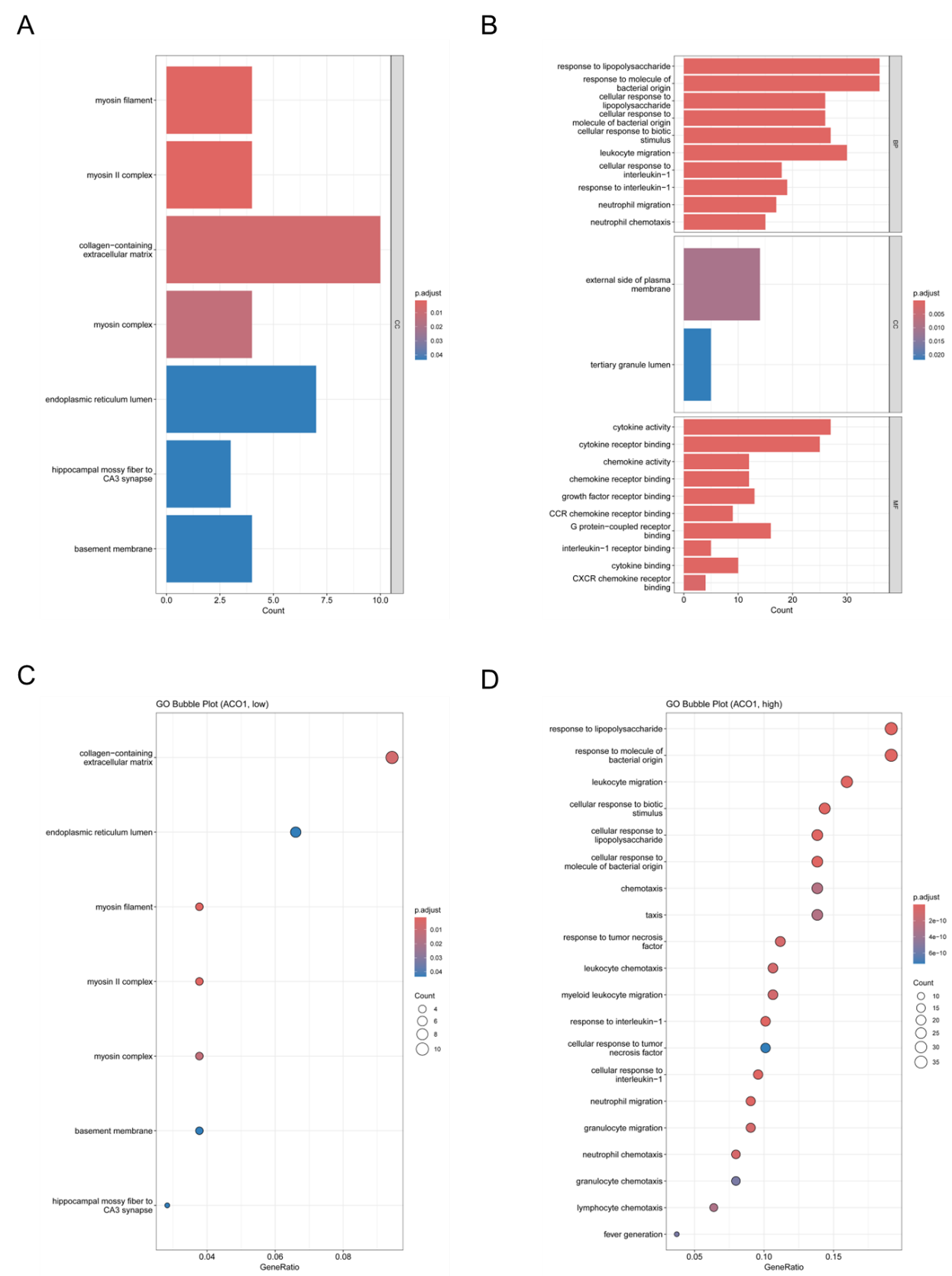

Supplement: Supplementary file 2 — Supplementary Material 2: Figure S2. GO enrichment and ssGSEA pathway analysis associated with ACO1 expression. (A) Bar plot of GO terms enriched in the low ACO1 expression group, mainly associated with extracellular matrix organization and structural components.;(B) Bar plot of GO terms enriched in the high ACO1 expression group, related to inflammatory cytokine response, chemotaxis, and immune activation.;(C) Bubble plot of GO enrichment for the low ACO1 group, showing significant enrichment in metabolic and structural pathways.;(D) Bubble plot of GO enrichment for the high ACO1 group, highlighting immune-related processes including granulocyte migration and cytokine-mediated signaling. [file 13048_2026_2036_MOESM2_ESM.png]

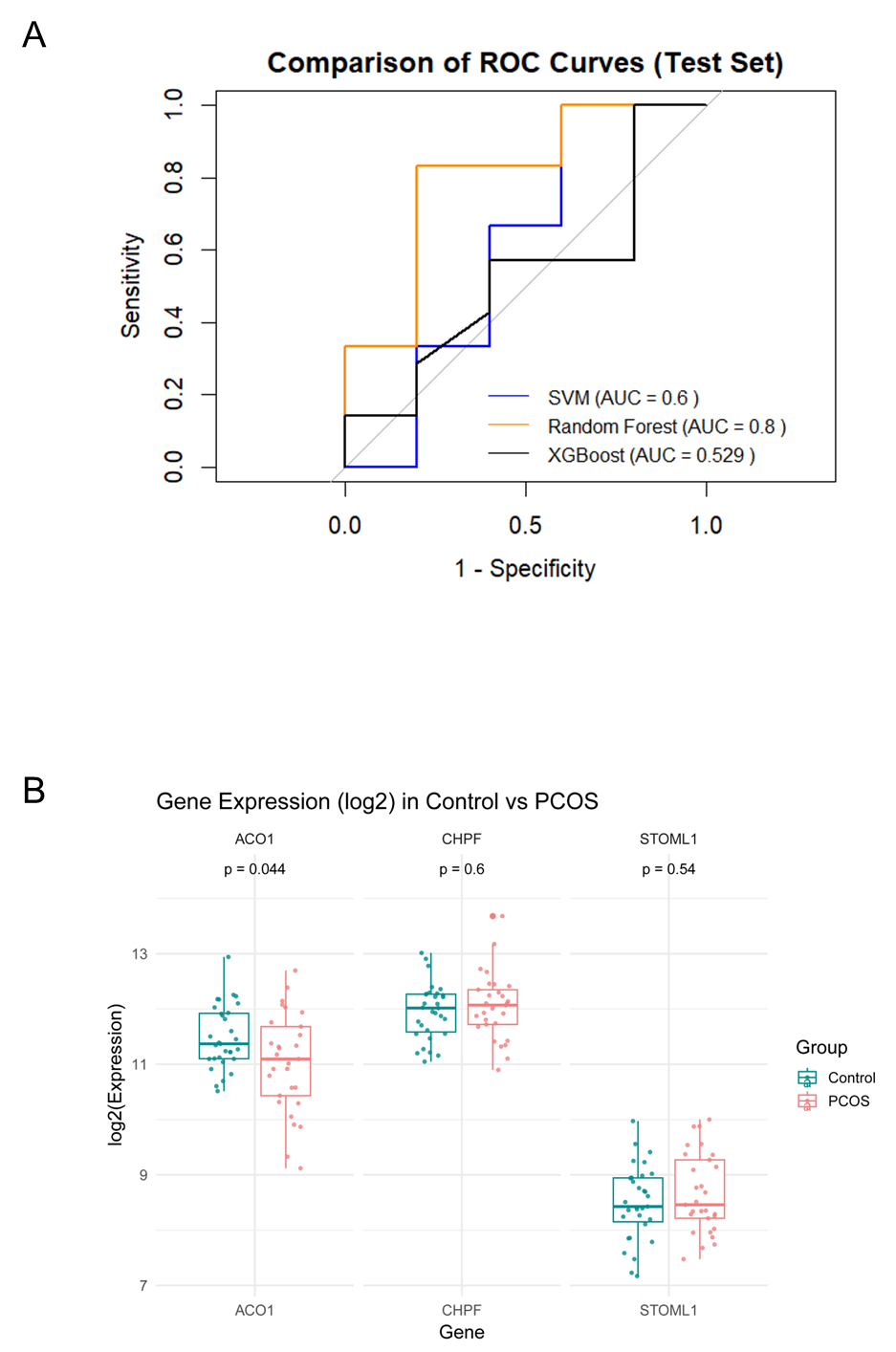

Supplement: Supplementary file 3 — Supplementary Material 3: Figure S3. Expression levels of core genes and model performance evaluation. (A) Receiver operating characteristic (ROC) curves and area under the curve (AUC) values of three machine learning models (SVM-RFE, Random Forest, XGBoost) in the validation cohort. The Random Forest model achieved the highest performance (AUC = 0.800).;(B) Box plots of log₂-transformed expression levels of ACO1, CHPF, and STOML1 in PCOS versus control samples. ACO1 showed a statistically significant difference between groups (p = 0.044). [file 13048_2026_2036_MOESM3_ESM.png]

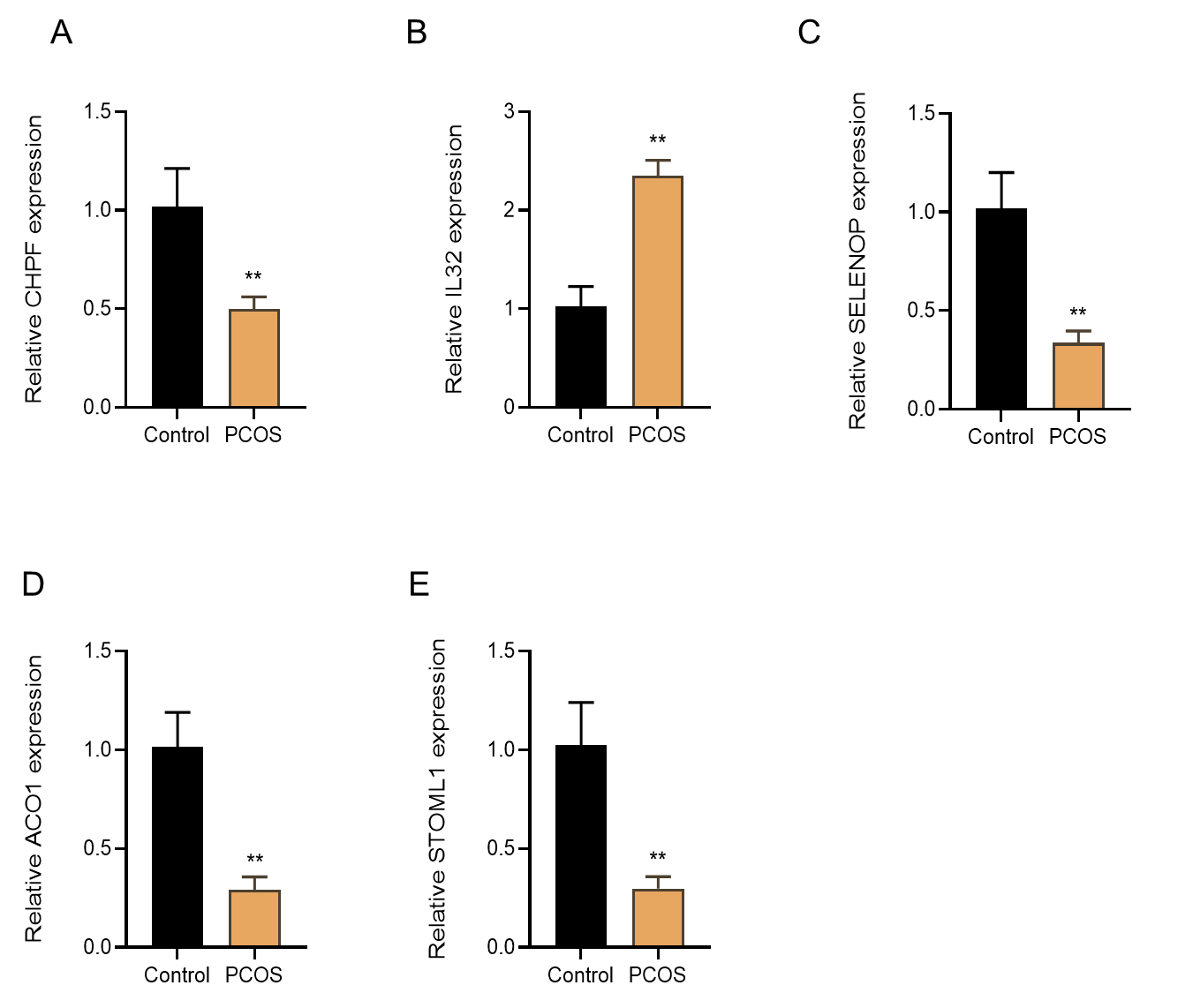

Supplement: Supplementary file 4 — Supplementary Material 4: Figure S4. qRT-PCR validation of core gene expression in clinical samples. Quantitative real-time PCR (qRT-PCR) was used to measure mRNA levels of the five core genes in granulosa cells from PCOS and control patients. CHPF, SELENOP, ACO1, and STOML1 were significantly downregulated in the PCOS group, while IL32 was significantly upregulated, consistent with transcriptomic findings. [file 13048_2026_2036_MOESM4_ESM.png]
