# Supplementary material for: An endometrial tissue-based predictive model for polycystic ovary syndrome constructed from immuno-metabolic dysregulation features mediated by ACO1
Source: J Ovarian Res. 2026 Feb 23;19:121. doi: 10.1186/s13048-026-02036-7 (PMC13036953; doi:10.1186/s13048-026-02036-7)
Supplement: Supplementary file 5 — Supplementary Material 5: Supplementary Tables. [file 13048_2026_2036_MOESM5_ESM.docx]

**Supplementary Table Legend**

**Table S1. Oligonucleotide primer sequences used for qRT-PCR validation.**

Forward (Fw) and reverse (Rv) primer sequences for five core genes (ACO1, STOML1, SELENOP, IL32, CHPF) are listed. Amplicon lengths are indicated.

| Name | | Sequence | Amplicon |
| --- | --- | --- | --- |
| ACO1 | Fw | TGGAGTGTGGTAGGAACACG | 151nt |
|  | Rv | ATCGAAAATGGTAAGCGCCC |  |
| STOML1 | Fw | GGCCTCATCAGTTTCCTGGG | 107nt |
|  | Rv | CGGAACACAATCATCCGCTC |  |
| SELENOP | Fw | TTGTGACAACCCCAGCAAT | 100nt |
|  | Rv | CATAAGGAGCTTTGGTCCTGG |  |
| IL32 | Fw | TCTCTCGGCTGAGTATTTGTGCC | 100nt |
|  | Rv | GGCCAAAAGTTCAAGGAGCCA |  |
| CHPF | Fw | CTGTGTCCTAAACCGGCTCG | 119nt |
|  | Rv | GTGCTGATGTAGCGGGTCC |  |

**Table S2. Performance metrics of the machine learning models for PCOS prediction.**

| **Model** | **Dataset** | **AUC** | **Sensitivity** | **Precision** | **Recall** | **F1** |
| --- | --- | --- | --- | --- | --- | --- |
| SVM | Train | 0.633 | 0.885 | 0.757 | 0.782 | 0.836 |
| SVM | Test | 0.567 | 0.800 | 0.649 | 0.633 | 0.716 |
| RandomForest | Train | 0.875 | 0.956 | 0.963 | 0.886 | 0.891 |
| RandomForest | Test | 0.800 | 0.950 | 0.793 | 0.783 | 0.727 |
| XGBoost | Train | 0.548 | 0.667 | 0.606 | 0.803 | 0.781 |
| XGBoost | Test | 0.559 | 0.613 | 0.557 | 0.747 | 0.635 |

**Table S3. Feature importance ranking derived from the random forest model.**

|  | MeanDecreaseAccuracy | MeanDecreaseGini |
| --- | --- | --- |
| ACO1 | 15.384 | 10.087 |
| CHPF | 4.431 | 7.874 |
| STOML1 | 5.191 | 4.113 |
